# Adaptive Micromixer Based on the Solutocapillary Marangoni Effect in a Continuous-Flow Microreactor

**DOI:** 10.3390/mi9110600

**Published:** 2018-11-16

**Authors:** Dmitry Bratsun, Konstantin Kostarev, Alexey Mizev, Sebastian Aland, Marcel Mokbel, Karin Schwarzenberger, Kerstin Eckert

**Affiliations:** 1 Department of Applied Physics, Perm National Research Polytechnic University, Perm 614990, Russia; DABracun@pstu.ru; 2 Institute of Continuous Media Mechanics, Perm 614013, Russia; kostarev@icmm.ru (K.K.); alex_mizev@icmm.ru (A.M.); 3 Faculty of Informatics/Mathematics, HTW Dresden, 01069 Dresden, Germany; sebastian.aland@htw-dresden.de (S.A.); marcel.mokbel@htw-dresden.de (M.M.); 4 Institute of Fluid Dynamics, Helmholtz-Zentrum Dresden-Rossendorf, 01328 Dresden, Germany; k.schwarzenberger@hzdr.de; 5 Institute of Process Engineering and Environmental Technology, TU Dresden, 01062 Dresden, Germany

**Keywords:** continuous-flow reactor, mixing, solutal Marangoni effect, relaxation oscillations

## Abstract

Continuous-flow microreactors are an important development in chemical engineering technology, since pharmaceutical production needs flexibility in reconfiguring the synthesis system rather than large volumes of product yield. Microreactors of this type have a special vessel, in which the convective vortices are organized to mix the reagents to increase the product output. We propose a new type of micromixer based on the intensive relaxation oscillations induced by a fundamental effect discovered recently. The mechanism of these oscillations was found to be a coupling of the solutal Marangoni effect, buoyancy and diffusion. The phenomenon can be observed in the vicinity of an air–liquid (or liquid–liquid) interface with inhomogeneous concentration of a surface-active solute. Important features of the oscillations are demonstrated experimentally and numerically. The periodicity of the oscillations is a result of the repeated regeneration of the Marangoni driving force. This feature is used in our design of a micromixer with a single air bubble inside the reaction zone. We show that the micromixer does not consume external energy and adapts to the medium state due to feedback. It switches on automatically each time when a concentration inhomogeneity in the reaction zone occurs, and stops mixing when the solution becomes sufficiently uniform.

## 1. Introduction

In the last decades, the interaction between reaction–diffusion phenomena and hydrodynamics has attracted increasing interests both from the fundamental point of view of nonlinear science and from application-oriented aspects in chemical engineering [1,2,3,4]. This arises from the fact that the chemically-induced changes of fluid properties such as density, viscosity, thermal conductivity or surface tension may result in flow instabilities, which exhibit a large variety of convective patterns.

From the technological point of view, continuous-flow microreactors are at the center of recent developments in chemical engineering [4]. Significant changes in this area were initiated in the early 2000s with the entrance of new technologies into organic synthesis [5]. Since the pharmaceutical production needs flexibility in reconfiguring the synthesis system rather than high throughput, increasingly miniature reactor design concepts are established [5,6]. This new reactor type provides several advantages with respect to the traditional batch-reactor. The latter in general is considered as a closed chamber without mass exchange between the reactor and the external environment. Hence, the different design and operation mode leads to following features:

- high productivity due to the absence of the loading-unloading stage, as well as cleaning of the reactor after each batch
- uniformity and stability of the process ensuring easy control
- stable consumption of reagents and energy due to the small volume of the reactor zone
- increase in output by the replication of the production line

This list is not intended to be exhaustive but exemplarily shows prominent benefits of this technology. Consequently, numerous studies have been published in recent years detailing the outcome of continuous-flow chemistry applied to single or multi-step syntheses of target compounds on various reaction schemes and spatial scales [7,8,9,10,11,12].

Two basic physical principles can be used to efficiently mix reagents in continuous-flow microreactors: diffusion and convection. When using diffusion as the main mixing tool, it is required to maintain high gradients of the diffusing components and to use channels with very small cross sections. Initially, the development of microreactor technology was mainly on this path [5,6]. With the miniaturization of the connecting capillaries, the flow remains laminar. These microreactors are manufactured without a particular reaction zone, since mixing of the reactants occurs directly after the junction of the feed capillaries. However, this approach has some drawbacks. For example, a significant pressure has to be applied to pump liquid through narrow channels involving higher energy costs. Simultaneously, the achievable reactor throughput is reduced. Further limitations arise due to the compatibility for different reaction kinetics. As the mixing time of reagents decreases, only fast reactions, i.e., small reaction times are possible. Other shortcomings, such as reactor fouling and high investment costs for precise manufacturing are discussed in the literature [13].

In the last few years, different types of microreactors and -mixers have been proposed [14,15,16,17]. Their operation principle relies not only on diffusion, but also on convective mixing. Microreactors of this type have a specifically designed reaction zone, where the flow forms vortex structures providing an efficient mixing of the reagents. This leads to a considerable increase in the product output and allows to run processes with lower reaction rates. Furthermore, various techniques have been developed for mixing typically laminar flows in narrow channels. Among them are passive methods where the mixing effect is introduced by geometry [18], e.g., using bas-relief structures on the bottom of the channel [19], but also active methods using an external Braille pin actuator array [20]. Further micromixer working principles are based on fluidic dielectrophoresis [21], or centrifugal action [22]. A recent theoretical study [23] investigates the collective behavior of hydrodynamically coupled molecular motors.

In this work, a recently discovered hydrodynamic effect is employed to create a novel, adaptive micromixer. For many years, the authors have been involved in the experimental and theoretical studies of the fundamental properties of soluto-capillary phenomena in different immiscible systems with [24,25,26,27,28,29,30,31] and without chemical reactions [32,33,34,35,36]. Among the various effects studied by the authors, a prominent finding was that solutal Marangoni convection frequently is accompanied by intensive relaxation oscillations. For example, this phenomenon can be observed in the vicinity of an air–liquid interface with inhomogeneous concentration of the soluble surfactant (aqueous solution of acetic acid) along the surface [33,35]. Similar oscillations were obtained in an immiscible liquid–liquid system, consisting of paraffin oil and water, with isopropyl alcohol as surface-active solute [36]. The mechanism of these oscillations was found to be a periodic coupling of the Marangoni effect, buoyancy convection acting in the presence of gravity and the restoring effect of diffusion. This interaction leads to a repeated consumption and regeneration of the Marangoni driving force. Because of that, a regular periodicity of convection is obtained. We now bring this particularly advantageous feature to a technological application.

Therefore, we show both experimentally and theoretically that the proposed micromixer can effectively work as a basic microreactor module. In this regard, the article is organized as follows. Section 2 presents different experimental observations of the relaxation oscillations at the interface of a single bubble and drop to underline the versatility of this effect with respect to various fluid combinations and geometries. Furthermore, we explain the mechanism of the oscillations used later as operation principle of the micromixer. The results of numerical simulations for a single droplet system are given in Section 3. Various aspects of the micromixer design are discussed in Section 4. A concrete implementation of our micromixer, based on a single bubble placed in the reaction zone, is also demonstrated here. Section 5 summarizes the results and provides some discussion.

## 2. Experimental Observations of Relaxation Oscillations

### 2.1. Liquid–Liquid System

In this section, we describe the main features of the basic hydrodynamic effect of the relaxation oscillations. The experimental system consists of two immiscible liquids placed in a Hele–Shaw cell as described below. The oscillations can be excited either near drops which formed during the filling procedure or near air bubbles produced via a capillary [36]. Here, we focus on the former case, while a second system with air bubbles is analyzed in Section 2.2.

#### 2.1.1. Experimental Setup

The liquid–liquid system consists of paraffin oil and water with 4.6 wt.% isopropyl alcohol being dissolved in the organic phase. The material parameters of this system are listed in [36]. All chemical substances are used as purchased. Water and isopropyl alcohol are of HPLC grade, and the paraffin oil is of pharmaceutical quality (Ph.Eur., DAB). The system is placed in a rectangular narrow cuvette of size L×H×W≈20mm×70mm×1mm by a special filling procedure described in [26,36] to satisfy the condition for the quasi 2D Hele–Shaw geometry. Due to its higher density, water is filled as the lower layer and paraffin oil with isopropyl alcohol forms the upper layer in the vertically oriented Hele–Shaw cell. We use a short-chain alcohol as surface-active solute to ensure that complex phenomena such as micelle formation or adsorption–desorption kinetics usually connected with strong surfactants only play a minor role. This justifies our simplified model employed in our simulations. Due to the hydrophilic nature of isopropyl alcohol, partition is largely in favour of the aqueous phase. This leads to an intense mass transfer and, accordingly, to a fast depletion of the organic phase in regions near the interface.

**Figure 1:**
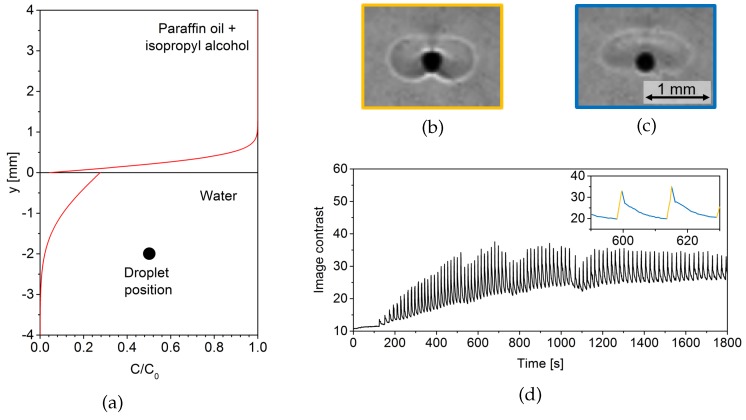
(**a**) The two-layer system paraffin oil/water with normalized concentration profile of isopropyl alcohol due to mass transfer out of the organic phase. The vertical position of a paraffin oil drop in the aqueous phase is marked by a black circle. (**b**) Active stage of relaxation oscillations at paraffin oil drop in the aqueous phase and (**c**) relaxation stage. (**d**) Time evolution of shadowgraph image contrast in the surrounding of the drop. The inset details two periods of relaxation oscillations.

The diffusive concentration profile, which establishes at the interface of the two layer system (material parameters according to [36]) after a time t=600 s is shown in Figure 1a as a function of the distance from the interface (y=0). The profile is calculated by the well-known analytical formula [37] assuming diffusion between two infinitely extended layers since the layer height (35 mm) is much larger than the distance of the concentration front to the interface. The concentration axis is normalized by the initial concentration in the donating paraffin oil phase. The graph clearly shows the depletion of the organic phase and the concentration profile developing in the lower aqueous phase. Note that the jump in the concentration profile at the interface is caused by the partition coefficient value 0.16 of isopropyl alcohol between paraffin oil and water.

During the filling procedure, drops of paraffin oil can form which remain at the wall of the Hele–Shaw cell in some distance of the interface. The vertical position of the drop, whose relaxation oscillations are plotted in Figure 1d, is marked by a black circle in Figure 1a. The diameter of the drops is typically 0.1–0.5 mm, corresponding to a volume of a few nanoliters. At these drops, pronounced relaxation oscillations occur as described next. To visualize the flow structure at the drops, a shadowgraph optics is employed (construction by TSO, Pulsnitz, Germany), operating in transmission. This technique detects the second derivative of the refractive index field. Hence, it qualitatively captures the patterns arising from interfacial convection since the convective mass transfer influences the concentration distribution and, consequently, the refractive index field.

#### 2.1.2. Experimental Results

Two characteristic stages of the relaxation oscillations are shown in Figure 1b,c. The corresponding drop of paraffin oil is immersed in the lower aqueous phase at a distance of 2 mm from the interface and sits at the wall of the Hele–Shaw cell. The droplet itself appears as a black circle in the shadowgraph optics due to its high interfacial curvature. In the surrounding of the droplet, the convective structure is visible as bright and dark regions differing from the quiescent fluid with intermediate gray value.

The periodicity of the oscillations can be described as follows. The cycle begins with the start-up of Marangoni convection in the form of two vortices at either side of the drop (this stage is shown in Figure 1b, orange frame). Due to the mass transfer of isopropyl alcohol across the overlying interface of the two-layer system, the drop is placed in a vertical concentration gradient (cf. Figure 1a). Hence, it is subjected to high solute concentration at the upper side, implying low interfacial tension since isopropyl alcohol makes the surface tension lower, and low concentration at the bottom, i.e., high interfacial tension. Therefore, the Marangoni convection at the drop surface is directed downwards, entraining fluid rich in solute to lower regions. This also affects the density stratification around the droplet. The initial density gradient is buoyantly stable because the solution density of the aqueous phase decreases with increasing isopropyl alcohol concentration. Due to the Marangoni-driven downflow, the stable density stratification locally is inverted. In the presence of gravity, this leads to a buoyancy-driven convection supporting the rise of the fluid in the Marangoni vortex at some distance from the interface. As a result, an intense mixing of the fluid, visible in Figure 1b, takes place which exhausts the vertical concentration gradient in the vicinity of the drop and leads to the breakdown of Marangoni convection.

Figure 1c (blue frame) displays the second characteristic stage, referred to as relaxation phase. During this stage, buoyancy-driven convection carries the mixed fluid upwards, and diffusion further redistributes the solute. After this, the conditions that existed before the active convective mass transfer around the drop are sufficiently restored. The vertical concentration gradient at the droplet again is high enough so that the system is ready to repeat the described cycle.

As visible in the comparison of Figure 1b,c, the intensity of convection in the surrounding of the drop influences the local contrast of the shadowgraph images. This is used in Figure 1d to illustrate the dynamics of the relaxation oscillations. The inset details the characteristic time evolution. The steep rise in image contrast (marked orange) corresponds to the short, impulsive stage of Marangoni convection. The relaxation phase (marked blue) lasts longer due to the slower processes of diffusion and buoyant convection being responsible for the restoration of the Marangoni driving force. The temporal overview shows that the oscillations start when the solute front, which penetrates the aqueous phase during mass transfer from the paraffin oil phase, reaches the droplet. The convection continues as long as the drop is subjected to sufficiently large concentration gradients. The oscillation frequency is highest at t≈400 s due to the passing solute front. When the aqueous solution near the interface is progressively equilibrated at later times, the concentration gradient diminishes and the oscillation frequency drops. This demonstrates that the mixing by Marangoni convection around such a drop or bubble automatically responds to the process state.

### 2.2. Air–Liquid System

In our second series of experiments, we examine the relaxation oscillations at a different configuration where an air–liquid interface is introduced in a vertical concentration gradient by a specifically designed setup. To further detail the fine structures in the velocity and concentration field during the relaxation oscillations, we now use a Fizeau interferometer for flow visualization.

#### 2.2.1. Experimental Setup

**Figure 2:**
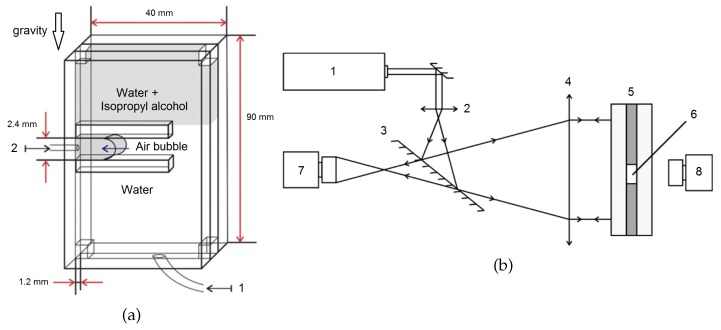
(**a**) Scheme of cuvette: the inlet for water during the filling procedure 1; the inlet for air to make the bubble 2. (**b**) Scheme of the Fizeau interferometer: helium-neon laser 1; microscope objective 2; semi-transparent mirror 3; objective-collimator 4; vertically oriented Hele–Shaw cell 5 with parallel glasses providing the workspace for interferometry; working chamber 6 with bubble or drop in channel; video cameras 7 and 8.

The cuvette for these experiments is again a vertically oriented Hele–Shaw cell with an adapted geometry to produce an air–liquid interface. The gap for the fluids is formed by two closely spaced parallel glass plates (Figure 2a). Inside the cuvette, a horizontal channel, which is 35 mm long, 2.4 mm high and 1.2 mm thick, is created by inserts. One end of the channel is open into the working chamber of the cell and the other is blocked off by the air bubble. Before the experiment, the cell is filled with water from the bottom inlet 1 and air is injected into the channel via the inlet 2 to produce the air bubble. The air bubble remains confined in the channel to prevent its buoyancy rise. Then, part of the water is discharged through pipe 1 up to the upper part of the horizontal channel and the vacant space above is filled with an aqueous solution of isopropyl alcohol of a given concentration. This results in the formation of a stably stratified two-layer miscible system consisting of pure water (bottom) and alcohol solution separated by a rather narrow initial diffusion zone. As a final step, a small amount of water again is discharged until the alcohol solution penetrates into the channel.

To visualize the concentration field, a Fizeau interferometer is employed (Figure 2b). In the resulting interferogram, the distribution of solute along a quasi-two-dimensional space (for example, the Hele–Shaw cell) is visualized as a system of interference fringes representing isolines of equal optical path length provided that the system is isothermal. If the concentration field can be assumed as two dimensional, i.e., concentration changes across the gap width can be neglected, then each interference fringe can be correlated to a certain concentration value. For our cell, a transition from one interference fringe to another corresponds to a change of isopropyl alcohol concentration in water by 0.27 wt.%. The interferometer can also be used to trace the evolution of flows in inhomogeneous solutions. Because of a very slow mass diffusion (the Péclet number is of the order of $10^{4}$), changes in the concentration distribution can be directly attributed to the fluid flow.

#### 2.2.2. Experimental Results

**Figure 3:**
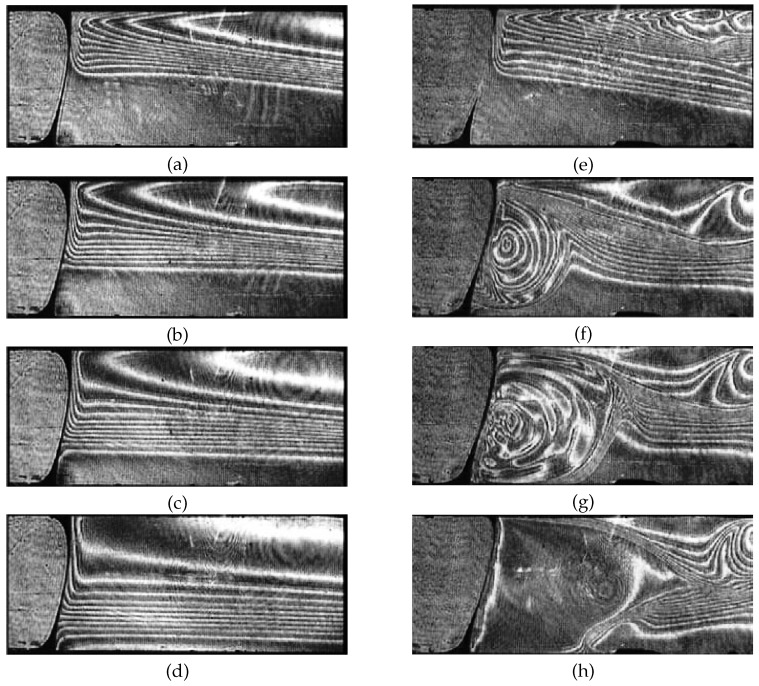
Evolution of the concentration field near the air bubble in the absence of pronounced convective mass transfer (**a**–**d**) for $C_{0}$ = 5 wt.% and at the onset of relaxation oscillations due to the coupling of solutal Marangoni convection with buoyancy and diffusion (**e**–**h**) for $C_{0}$ = 6 wt.%. The left and right frames from up to down correspond to time *t*: 0 s (**a**); 13 s (**b**); 30 s (**c**); 105 s (**d**); 0 s (**e**); 15.3 s (**f**); 17 s (**g**); and 22 s (**h**).

Figure 3 displays a series of interferograms describing the evolution of the concentration field near the air bubble with an initial concentration of the isopropyl alcohol solution $C_{0}$. The interferograms show a part of the channel in the Hele–Shaw cell sketched in Figure 2a. One can see that the aqueous solution of alcohol gradually advances towards the bubble in the form of a tongue (see, for example, Figure 3a), which finally touches the bubble surface in the upper part. Thereby, it creates a surface tension difference, i.e., a Marangoni stress along the air–liquid surface, providing the conditions for the onset of Marangoni convection.

The experiments revealed that a minimum concentration gradient is required for the onset of pronounced interfacial convection. Hence, the flow only is amplified when the resulting concentration difference at the interface is higher than a certain threshold value $▵C^{*}$. Figure 3 illustrates this by comparing snapshots of the time evolution near this threshold. The left-hand column of interferograms corresponds to an initial concentration of $C_{0}$ = 5 wt.% resulting in a concentration difference ▵C, which is slightly lower than the threshold value $▵C^{*}$. A slightly higher concentration $C_{0}$ = 6 wt.% is used for the snapshots in the right-hand column which turned out to be enough to excite the convection. The existence of a clear threshold for the onset of convection indicates the presence of adsorbed trace impurities on the surface which cannot be fully excluded despite our rigorous cleaning procedure. Such surface-active impurities can change the surface rheology or can induce counteracting Marangoni stresses when redistributed by an arising interfacial flow. Thus, to initiate interfacial convection, it is necessary that the Marangoni shear stress by the acting concentration gradient is high enough to overcome these effects. This is the case for the experiment at $C_{0}$ = 6 wt.% when the tongue of the alcohol solution approaches the bubble surface (Figure 3e). An intensive Marangoni flow appears immediately after the tongue touches the bubble surface at a time t=15.28 s. In contrast to that, for $C_{0}$ = 5 wt.%, the convection cannot develop even 105 s after the contact with the alcohol tongue (Figure 3d). In the latter case, the downward propagation of the solute front mainly is caused by diffusion.

Generally, the development of the convection near the bubble for $C_{0}$ = 6 wt.% is similar to the scenario described in Section 2.1. At the first stage, the fluid flow is directed downward along the bubble surface (the vortex rotates counterclockwise), propagating into the region of lower isopropyl alcohol concentration (Figure 3f). Over time, the vortex entrains more and more solute-rich fluid to the lower region (Figure 3g), decreasing the density in comparison to the surrounding. As a result, the lighter liquid begins to rise upward due to buoyancy pushing the alcohol tongue away from the bubble (Figure 3h). The last stage is responsible for the restoration of the Marangoni driving force.

**Figure 4:**
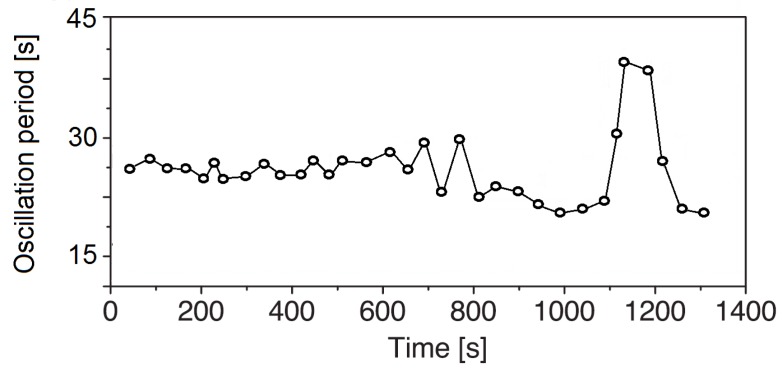
Time evolution of the oscillation period for an aqueous isopropyl alcohol solution at $C_{0}$ = 6 wt.%.

Figure 4 shows the time evolution of the oscillation period. Due to the intensive stirring of the solution near the bubble, the regeneration time for the influx of fresh isopropyl alcohol solution increases, thus longer oscillation periods can be observed in the later stage of the experiments.

## 3. Numerical Simulations

### 3.1. Mathematical Model

Numerical simulations of the flow around drops or bubbles in a concentration gradient of a surface active solute allow us to estimate the influence of specific material constants by a defined parameter variation. We use a diffuse interface model, whereby the governing equations are formulated according to the Hele–Shaw approximation [25,27,38,39]. The Hele–Shaw approximation assumes that the fluids are enclosed in the gap between two parallel plates with a small distance *d* such that the fluid motion becomes mainly two-dimensional. In line with this, the drop/bubble is modeled as a cylindrical fluid domain between the plates.

In the surrounding aqueous phase, an initially constant vertical concentration gradient of isopropyl alcohol is supposed. That means the non-steady state in the experiment with an evolving concentration profile in the aqueous phase is approximated by a steady-state concentration field with a constant concentration gradient. The solute concentration in the drop/bubble is neglected. According to the diffuse interface model, a phase field ϕ is used to represent the phases, with ϕ=0 in the drop/bubble and ϕ=1 in the surrounding fluid. We assume diffusion-limited adsorption where local thermodynamic equilibrium exists between the excess concentration Γ at the interface and the solute concentration *C* adjacent to the interface. A linear relation between both quantities given by the Henry isotherm Γ=KC (*K* stands for the adsorption coefficient) is applied so that interfacial tension σ as well depends linearly on *C*. The mass transport is modeled by an advection–diffusion equation and the momentum transport by the incompressible Navier–Stokes equations coupled with the shear stress balance at the interface. The resulting governing equations for velocity V, pressure *p* and solute concentration *C* in the aqueous phase are based on [36,40]:

(1) $$\begin{array}{c} \rho \left(\phi ,C\right)\partial_{t}V-\nabla \cdot \left(\eta \left(\phi \right)\left(\nabla V+\nabla V^{T}\right)\right)+\frac{3}{d^{2}}\eta \left(\phi \right)V+\nabla p+k\nabla \phi ⊗\nabla \phi \cdot V=\\ \;\;\;\;\;\;g\rho \left(\phi ,C\right)+\sigma_{ref}\alpha_{C}\left|\nabla \phi \right|P\nabla C, \end{array}$$

(2) $$\begin{array}{c} \nabla \cdot V=0, \end{array}$$

(3) $$\begin{array}{c} \phi \partial_{t}C+\phi V\cdot \nabla C-D\nabla \cdot \left(\phi \nabla C\right)-D_{n}\nabla \cdot \left(\left(n\cdot \nabla C\right)|\nabla \phi |n\right)=0, \end{array}$$

where g is the gravitational acceleration, $\sigma_{ref}$ is the interfacial tension at zero solute concentration, $\alpha_{C}$ is the interfacial tension coefficient, *D* is the diffusion coefficient of isopropyl alcohol in the aqueous phase, P=I−n⊗n is the operator of the surface projection, I is the identity matrix, n=∇ϕ/|∇ϕ| is the outer normal to the drop and *k* is a penalty constant enforcing zero normal velocity at the surface. $D_{n}$ stands for an interfacial normal diffusion ensuring the desired no flux boundary condition at the drop surface. The third term on the left-hand side of Equation (1) depending on the gap width *d* is introduced due to the Hele–Shaw approximation. Assuming a parabolic velocity profile between the plates, it accounts for the friction at the walls. Linear interpolations are used for the density ρ and viscosity η in the phase field model:

(4) $$\begin{array}{c} \rho \left(\phi ,C\right)=\phi \rho_{ref}^{\left(1\right)}\left(1-\beta_{C}^{\left(1\right)}C\right)+\left(1-\phi \right)\left(\rho_{ref}^{\left(2\right)}\left(1-\beta_{C}^{\left(2\right)}C\right)\right), \end{array}$$

(5) $$\begin{array}{c} \eta \left(\phi \right)=\phi \left(\eta^{\left(1\right)}\right)+\left(1-\phi \right)\eta^{\left(2\right)}, \end{array}$$

where (i) denotes the fluid phase with i=1 for water and i=2 for paraffin oil/air, $\beta_{c}$ is the volume expansion coefficient and $\rho_{ref}$ is the density at zero solute concentration.

Simulations are performed for a set of material parameters corresponding to an aqueous solution of isopropyl alcohol surrounding a paraffin oil drop, whereas simulations for an air bubble are shown in [36]. The drop diameter is set to 0.3 mm which is a typical value according to the experimental observations in Section 2.1. The domain size was chosen $L_{x}\times L_{y}$ = 8 mm× 8 mm which was verified to be large enough to eliminate finite-size effects. The grid resolution is adapted to capture the fine structures arising during Marangoni convection. The mesh size (longest side of triangles) amounted to $3.906\times 10^{-5}$ m at the interface and $1.5625\times 10^{-4}$ m in the bulk phase. An initial concentration gradient of dC/dy = 25 mmol/(l·mm) is estimated from the diffusive concentration profile (Figure 1a) in the aqueous phase near the interface at a time of 600 s. For the velocity, the initial and boundary condition is set to V = 0. This quiescent initial state is unstable, since the vertical concentration gradient imposes interfacial tension gradients at the drop surface and Marangoni convection sets in as already observed experimentally. Since the experiments indicate that the buoyancy-driven convection plays an important role for the mechanism of the relaxation oscillations, the volume expansion coefficient in the aqueous phase $\beta_{C}^{\left(1\right)}$ is varied in relation to the reference value of isopropyl alcohol in steps of 0.1, 0.25, 0.5, 1, 2, 4, 10. This corresponds to solutes which have a weaker or stronger influence on the solution density in comparison to isopropyl alcohol. Note that the highest values of 4 and 10 rather are of theoretical interest since they strongly exceed the typical volume expansion coefficients of aqueous solutions. Furthermore, we only consider systems with density-lowering solutes, i.e., negative volume expansion coefficients. Otherwise, the system dynamics would be governed by Rayleigh–Taylor instability in the aqueous phase.

### 3.2. Numerical Results

**Figure 5:**
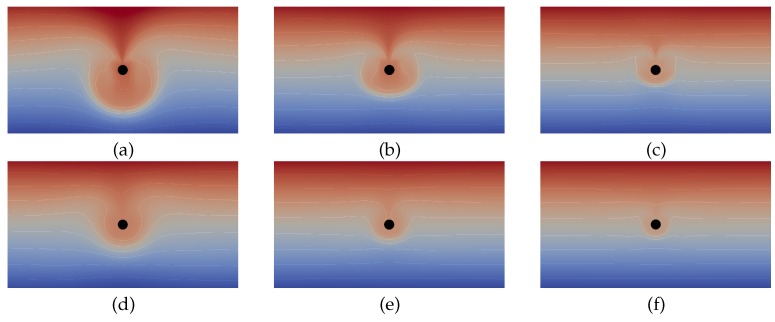
Concentration distribution from simulations of a paraffin oil drop placed in an aqueous solution with a vertical gradient of surface active solute. The value of the volume expansion coefficient is varied relative to that of isopropyl alcohol as: 0.1 (**a**,**d**); 1 (**b**,**e**); and 10 (**c**,**f**). Shown is a section of 5.5 mm× 3 mm of the computational domain. The upper row (**a**–**c**) displays the active stage of Marangoni convection, corresponding to a local maximum in the curves of Figure 6b, while the lower row (**d**–**f**) shows the final part of the relaxation stage, corresponding to a local minimum in the curves of Figure 6b.

**Figure 6:**
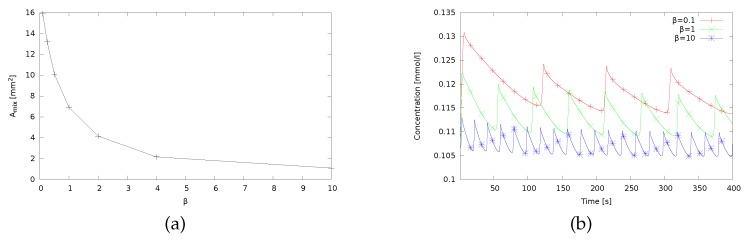
(**a**) Area of mixed fluid directly after the termination of the active stage of Marangoni convection from numerical simulations as a function of the volume expansion coefficient. (**b**) Relaxation oscillations of the mean solute concentration at the drop surface for different values of the volume expansion coefficient.

Figure 5 shows snapshots of the concentration distribution around the drop for increasing magnitudes of the volume expansion coefficient $\beta_{C}^{\left(1\right)}$ from left to right. The stage of active Marangoni convection is displayed in the upper row and the end of the relaxation stage is depicted in the lower row. The observed structure of the concentration distribution agrees with the mechanism of the relaxation oscillations discussed in Section 2. In the active stage, one can clearly see the strong inflow of solute-rich fluid at the top of the drop and the mixing zone which encloses the drop and finally extinguishes the driving concentration gradient. With a higher magnitude of the volume expansion coefficient, the density stratification in the bulk fluid becomes increasingly stabilizing. This implies that more work is necessary to shift a fluid element vertically by the Marangoni convection what counteracts the mixing effect. As visible in Figure 5, the zone of the mixed fluid strongly shrinks from left to right. This effect is quantified in Figure 6a by plotting the mixing area $A_{mix}$ directly after the termination of the active stage over the relative volume expansion coefficient (again normalized by the value for isopropyl alcohol as the reference case). We define $A_{mix}$ as the area where the concentration *C* deviates more than 5 mmol/L from the initial linear concentration profile. The curve in Figure 6a shows that $A_{mix}$ tends to zero for very high magnitudes of the volume expansion coefficient, but strongly increases if this value is small.

The buoyancy effects influence not only the structure of the convection, but also the oscillation dynamics. To characterize the time evolution, the mean solute concentration

(6) $$\begin{array}{c} \langle C\rangle =\frac{1}{2\pi r}\int_{\Omega }\left|\nabla \phi \right|Cdx, \end{array}$$

at the drop surface Ω is shown in Figure 6b for varied volume expansion coefficients. Since the initial concentration gradient is constant throughout the whole domain, the first cycle usually is more intense, while the subsequent periods start from a pre-mixed state. It can be seen that, for a low magnitude of the volume expansion coefficient (almost buoyantly neutral solute), the curve oscillates around a higher concentration value. Without hindrance by a stabilizing density stratification, a more effective inflow of solute-rich fluid from the upper regions can take place. In the quiet relaxation phase, the concentration at the interface only drops to a moderate value. Furthermore, it takes longer to redistribute the larger mixing area so that the oscillation frequency decreases. When the relative volume expansion coefficient is high, the stronger buoyancy quickly restores the vertical concentration gradient and the next short oscillation begins.

The order of magnitude for the oscillation frequency in the simulations satisfactorily agrees with the experiments considering the simplifications inherent in the theoretical model. Besides the applied linearizations for the dependence of interfacial tension and solution density on isopropyl alcohol concentration, and general uncertainties in the material properties, the 2D Hele–Shaw approximation is the main source for the quantitative deviations observed. Since the size of the drop (0.3 mm) is significantly smaller than the gap width of the Hele–Shaw cell (1 mm), only one side of the drop touches the wall in the experiments, whereas the Hele–Shaw approximation assumes a cylindrical form of the drop between the glass plates. Therefore, significant 3D effects have to be taken into account, which certainly influence the temporal evolution. However, as shown above, our theoretical approach allows us to study the influence of relevant material parameters and to obtain qualitative trends with the advantage of a strongly reduced computational cost compared to full 3D simulations.

## 4. Micromixer Using a Single Bubble Inside the Reactor

**Figure 7:**
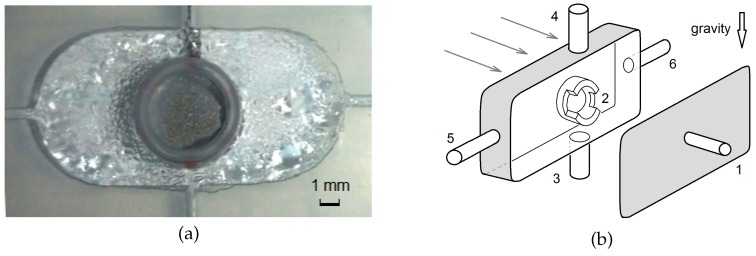
(**a**) Photograph as general view of the reaction zone of the continuous-flow microreactor with an adaptive mixing effect due to an air bubble placed in the center. (**b**) Scheme of the cuvette: inlet for air to produce the bubble 1; plastic insert to hold the bubble 2; inlets for the reagents 3 and 4; outlets for the reaction product 5 and 6. Arrows indicate the direction of observation with the Fizeau interferometer.

By taking into account the characteristic properties of the relaxation oscillations discussed in the preceding sections, we propose a novel design of a continuous-flow microreactor based on the mixing effect of the solutal Marangoni convection. The general view and the internal details of the reactor are shown in Figure 7a,b. The working area of the reactor is a quasi-rectangular cell placed between two parallel glasses. A PTFE liner between the glass plates sets the inner dimensions of L×H×W=20mm×10mm×2 mm. The narrow gap between the glass plates again provides quasi two-dimensionality of the fluid flow. The reactor was used in a vertical position so that the gravity vector is parallel to the front and back glass plates (Figure 7b).

To switch on the Marangoni effect inside the reactor, a free surface is necessary. For this purpose, we have placed a plastic ring with a diameter of 6 mm in the middle of the cavity. This ring bounds the area in which the air bubble is created and holds the bubble in place during convective mixing of the fluid. To ensure sufficient contact of the bubble with the reacting fluids, symmetrical cutouts are inserted in the ring as shown in Figure 7b. By this, the bubble is fixed between solid walls from above and below, but the lateral bubble surface is free. The depth of the notch in the plastic ring is 1 mm, i.e., half the gap width between the glass plates. Each of the lateral notches has an angular dimension of $150^{\circ }$. In the middle of one of the glass plates, tube 1 is inserted, through which air can be supplied to form a bubble inside the circular cell 2. The bubble can be retained in the cell up to a feed inflow velocity of 1 mm/s. When this critical pumping rate is exceeded, the flow can detach the air bubble from the holding ring. Since flow visualization inside the air bubble is not required, cell 2 is covered by an opaque moisture-repellent film on both sides.

In the reactor, two vertical channels (3 and 4) are provided, through which the reagent solutions are fed (Figure 7b). The two horizontal channels (5 and 6) serve to withdraw the reaction products from the reactor zone. Such a reactor design can be used, for example, to implement a quite common second-order scheme A + B → C, given that one of the substances is surface-active. To test the operation of the mixer, a non-reacting system is used. The procedure to prepare the device for operation is as follows. At the beginning, the cavity is completely filled with water through tube 3. Then, the air bubble is injected in the cell 2 via tube 1. At the next stage, an aqueous solution of isopropyl alcohol, which serves as a working surfactant, is continuously fed into the reactor cavity through tube 4 concurrently with the inflow of pure water from below.

**Figure 8:**
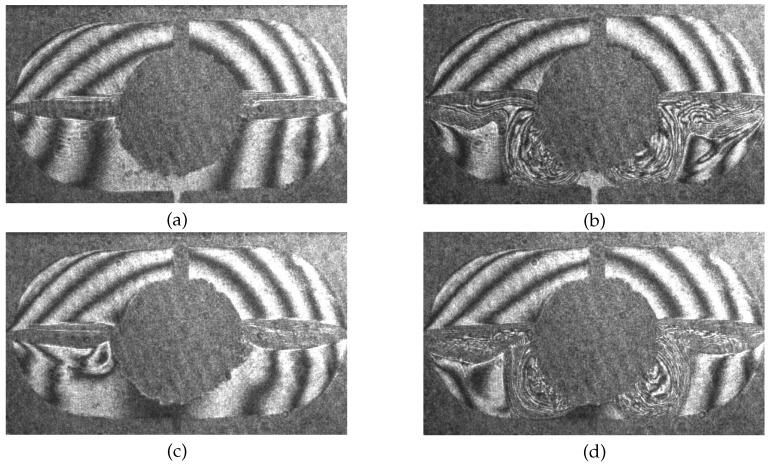
Evolution of the concentration field of isopropyl alcohol near the air bubble placed in the center of the cavity for $C_{0}$ = 15 wt.% and inflow velocities from both inlets *V* = 0.02 mm/s: *t* = 240 s (**a**); *t* = 270 s (**b**); *t* = 420 s (**c**); and *t* = 450 s (**d**).

Figure 8a–d shows four snapshots of the time evolution in the reactor at the onset of the relaxation oscillations. At the very beginning, there is a stage without intense Marangoni convection along the free surface of the bubble. Consequently, the liquid leaves the reactor via side channels 5 and 6 rather unmixed: the vertical concentration stratification is clearly visible in Figure 8a. This induction period probably is connected to the threshold of Marangoni convection as noted above. At *t* = 250 s the active phase is initiated and the fluid is mixed by the intense Marangoni convection (Figure 8b). The first stage of vigorous convective motion lasts about 150 s. This prolongation of the active phase can be explained by the fluid flow which is superposed by the external pumping. It continuously feeds solute-rich fluid supporting the Marangoni vortex at the bubble.

When the solution in the reactor zone becomes completely mixed so that the concentration difference on the surface of the bubble strongly decreases, the relaxation phase occurs. Nevertheless, according to the structure of the interferogram stripes, one can conclude that a weak convective movement still continues, and the mixed liquid leaves the reactor via lateral channels (Figure 8c). During the relaxation phase, fresh alcohol solution is supplied from the top channel again creating the concentration difference on the bubble surface. The relaxation phase lasts for a relatively short time (approximately 20–30 s). Thereafter, the Marangoni convection is excited again, as shown in Figure 8d. Interestingly, the mixing area is located in the lower part of the reactor. Most likely, this is caused by the interaction of the Marangoni convection with the concentration stratification and the superposed feed flow. This is an important observation for optimizing the reactor design in future work.

## 5. Discussion and Conclusions

In this work, we propose a new micromixer design based on the joint action of solutal Marangoni instability, buoyancy convection and diffusion. For this function principle, the presence of a fluidic interface and a surface-active solute is required. The operation of the micromixer does not consume additional external energy, but works due to the repeated consumption and regeneration of the driving concentration gradients. Thus, the micromixer automatically adapts to the conditions in the reactor zone. If the concentration field becomes sufficiently uniform, the oscillations stop. When the concentration inhomogeneities reappear, the system indicates that the reagents in the reactor zone are still not completely mixed and the convection is excited again. For specific applications in chemical engineering, this adaptability will be advantageous, e.g., for the degradation of pollutants, where higher educt concentrations require an intensified process. For common continuous-flow microreactors, where fresh reactants are permanently delivered into the mixing zone, the concentration gradient will be constant in time, providing the necessary inhomogeneity around the bubble. Furthermore, a continuously operating micromixer independent of the medium can be obtained by introducing an inert surfactant (not participating in the reaction) in the feed flow.

Indeed, the application of such a microreactor requires an individual approach to the specific reaction type and the used solutes. Our experiments however show that the relaxation oscillations occur in different geometries and fluid combinations making it versatile for the implementation in various microreactor systems. The formation of bubbles is an inexpensive method to introduce a fluidic interface in a microreactor setup. On the other hand, the liquid–liquid system is relevant for droplet microfluidics where the droplets themselves are used as a spatially bounded reaction zone. Our tested micromixer also works in the presence of slight contaminations if the Marangoni driving force is high enough, which is important under technological conditions.

However, further developments are necessary to approach technological applicability. Our simulations support this by providing a thorough understanding of the basic mechanisms and dependencies of the relaxation oscillations. They suggest that an even more effective mixing can be obtained for solutes with lower volume expansion coefficient or under a reduced influence of gravity, i.e., in a horizontal reactor position. The identification of such determining parameters gives the directions for future experimental work.
